# Chronic Lymphocytic Leukemia: Time-Limited Therapy in the First-Line Setting and Role of Minimal Residual Disease

**DOI:** 10.1007/s11912-023-01482-6

**Published:** 2024-01-04

**Authors:** Janina Stumpf, Othman Al-Sawaf

**Affiliations:** 1 University of Cologne, Faculty of Medicine and University Hospital Cologne, Department I of Internal Medicine, Center for Integrated Oncology Aachen Bonn Cologne Duesseldorf, Cologne, Germany; 2 Cancer Institute, University College London, London, UK; 3 Francis Crick Institute, London, UK

**Keywords:** MRD, Minimal residual disease, CLL, Chronic lymphocytic leukemia, Fixed duration, Time-limited therapy

## Abstract

**Purpose of Review:**

In this review, we provide an overview of different time-limited combination therapies of chronic lymphocytic leukemia (CLL) and summarize the data of pivotal clinical studies. Furthermore, we discuss the relevance of MRD in clinical trials and summarize the challenges that arise in routine clinical care. Finally, we provide an outlook on studies and datasets needed to optimize the use of time-limited treatment strategies and MRD assessments in modern CLL management.

**Recent Findings:**

In recent years, first-line treatment of CLL has undergone a considerable transformation, with targeted substances having largely replaced chemoimmunotherapy (CIT) as a time-limited strategy in the frontline setting. BTK inhibitors were the first class of targeted agents introduced in CLL, which achieved longer progression-free survival (PFS) and in some cases also overall survival (OS) than CIT. However, this required an indefinite drug intake until disease progression, while CIT is generally administered over the course of few months. In contrast to BTK inhibitors, BCL2 inhibitors, another class of targeted agents, can achieve high rates of undetectable minimal residual disease (uMRD) levels and induce deep molecular remissions with the potential to stop treatment while maintaining remissions.

**Summary:**

Combinations of BCL2 inhibitors with CD20 antibodies or with BTK inhibitors have been explored to establish time-limited treatment strategies with targeted agents. In this context, one of the strongest predictors of long-term outcomes is MRD status at the end of treatment, which has been shown to correlate closely with PFS and OS in most cases.

## Introduction

Time-limited treatment of chronic lymphocytic leukemia (CLL) has been the standard-of-care for decades: The first chemotherapy-based regimens, such as fludarabine, cyclophosphamide (FC), or FC rituximab (FCR), were designed as fixed-duration regimens that were administered for six cycles [1–3]. While this strategy allowed patients to benefit from long treatment-free intervals, certain subgroups, particularly patients with *TP53* dysfunction or an unmutated IGHV gene status, experienced reduced treatment efficacy [4]. Targeted inhibitors of the B-cell receptor (BCR) signaling pathway, in particular inhibitors of Bruton tyrosine kinase (BTK), have demonstrated high efficacy across all prognostic subgroups of CLL and thus have largely replaced chemoimmunotherapy [5, 6]. However, this treatment strategy requires a continuous, indefinite treatment until non-tolerance or disease progression, which can entail many years of daily drug intake [7]. This can be associated with cumulative toxicities, impaired quality of life, and a considerable health-economic burden [8]. In contrast, time-limited therapies might have less treatment-related toxicity and side effects and lower rate of clonal evolution and resistance mutation, since drug intake is only required for a limited number of cycles [9].

To date, the achievement of uMRD after treatment discontinuation predicts long-lasting remissions. In the context of chemo- and chemoimmunotherapy, patients with uMRD had a significantly longer progression-free survival (PFS) than patients with detectable MRD (dMRD) [2, 10–12]. With BTK inhibitor monotherapy via agents like ibrutinib, fewer than 10% of patients reach uMRD, thus warranting continuous treatment to achieve disease control [13]. With venetoclax monotherapy, up to 40% of patients reach uMRD levels in the relapsed/refractory setting [14, 15]. Hence, targeted combination regimens using a BCL2-inhibitor backbone have become one of the cornerstones of modern CLL management: Currently, the combination of the BCL2 inhibitor venetoclax and the CD20 antibody obinutuzumab are FDA and EMA approved as well as the all-oral combination of venetoclax with the BTK inhibitor ibrutinib (only EMA approved) for the first-line treatment of CLL.

In this review, we summarize the development of time-limited targeted treatment strategies of CLL and break down the data of pivotal clinical studies that established modern time-limited treatment of CLL. Moreover, we discuss the relevance of MRD for research purposes and its potential to further personalize time-limited treatment of CLL.

## Time-Limited First-Line Treatment of CLL

Currently, two time-limited, targeted treatment regimens are approved, both of which use the BCL2 inhibitor venetoclax as a backbone. In 2019, the CLL14 study demonstrated for the first time that a combination of venetoclax and the CD20 antibody obinutuzumab was associated with longer progression-free survival in patients with previously untreated CLL and coexisting disease, compared with chlorambucil-obinutuzumab [16]. In this randomized phase 3 trial, 432 unfit patients, as defined by CIRS > 6 and/or eGFR between 30 and 69 mL/min, were randomized 1:1 to receive either 12 cycles of venetoclax-obinutuzumab (VO) or chlorambucil-obinutuzumab (ClbO). Patients with *TP53* deletion or mutation could be included at the investigator’s discretion. Two years after treatment cessation, with a median follow-up of 39.6 months (IQR 36.8–43.0), patients treated with VO had significantly longer PFS than patients with ClbO (HR 0.31, 95% CI 0.22–0.44; *p* < 0.0001) [17]. Median PFS was not reached in the VO group vs. 35.6 months (33.7–40.7) in the ClbO group. The combination of VO remained superior to chemoimmunotherapy in terms of PFS, but no statistically significant difference in overall survival (OS) was observed (HR 0.85; 95% CI, 0.54 to 1.35; *p* = 0.49) [18, 19]. Further follow-up analyses of this study demonstrated that patients with del(17p) and/or *TP53* mutation had a longer PFS when treated with VO compared to ClbO (5-year PFS 40.6% vs 15.6%; HR 0.48, 95% CI 0.24–0.94) [20••]. However, patients with del(17p) and/or *TP53* mutation had a shorter PFS than patients without, regardless of the treatment arm. In both study arms, patients with unmutated IGHV status had a shorter PFS, and the simultaneous presence of del(17p) and/or *TP53* mutation unmutated IGHV status was associated with the shortest PFS. Regarding overall survival, patients with del(17p) and/or *TP53* mutation had a shorter OS than patients without del(17p) and/or *TP53* mutation in both arms (VO/ClbO). Patients with unmutated IGHV status treated in the ClbO arm had significantly shorter OS than patients with mutated IGHV status; no difference was shown for VO. These data confirm the long-term benefit of the 1-year VO regimen in elderly, unfit patients with previously untreated CLL. Several phase 1 and phase 2 studies reported similar outcomes with VO, with response rates approaching 100% in treatment-naïve patients and 95% in relapsed/refractory patients [21, 22].

Recently, the analysis of the randomized CLL13 study showed comparable results for venetoclax-based regimens in fit patients [23••]. In this phase 3 trial, 926 fit patients without *TP53* aberration were assigned in a 1:1:1:1 ratio to the treatment arms CIT (FCR or bendamustine-rituximab (BR)), venetoclax/rituximab (VR), venetoclax/obinutuzumab (VO), or venetoclax/obinutuzumab/ibrutinib (VOI). Three-year progression-free survival was highest for the triple combination with VOI (90.5%), closely followed by the dual combinations VO (87.7%) and VR (80.8%), and 75.5% in the chemoimmunotherapy arm. In the subgroup with unmutated IGHV, there was primarily a PFS benefit for patients treated with VOI (86.6%) and VO (82.9%) versus 65.5% for CIT; no significant differences were observed in the group with mutated IGHV. No differences were observed in overall survival (OS).

Overall, these data provide robust evidence on the efficacy of the fixed-duration VO regimen in young and fit as well as elderly and unfit patients with treatment-naïve CLL. Most national guidelines have therefore implemented VO as an option for first-line CLL [24–26].

Fewer randomized data are available on the combination of venetoclax and BTK inhibitors as an all-oral fixed-duration combination. In several phase 2 studies, both in the first-line and the relapsed/refractory setting, the combination of VI showed ORR of 96% and uMRD rates after approximately 1 year of combination treatment of 56% [27, 28]. The phase 2 CAPTIVATE study has so far generated the largest prospective dataset on fixed-duration VI in the first-line setting: In a cohort of 159 patients, 136 of whom did not have del(17p) and a mean age of 60 years, the estimated 24-month PFS rates after a median follow-up of 27.9 months were 95% (95% CI 90– 97) in the overall treated population, 96% (95% CI 91– 98) in patients without del(17p), and 84% (95% CI 63– 94) in patients with del(17p)/mutated *TP53* [29]. The estimated 24-month OS rates were 98% (95% CI 94– 99) in the overall population, 98% (95% CI 93–99) in patients without del(17p), and 96% (95% CI 76–99) in patients with del(17p)/mutated *TP53*. Combination therapy with ibrutinib plus venetoclax for 12 cycles results in clinically meaningful PFS and treatment-free remissions in patients with previously untreated CLL, including patients with high-risk disease features [30]. The only randomized data on first-line VI were generated in the GLOW study, which compared VI to ClbO in elderly and unfit patients [31, 32]. In total, 211 patients were randomized 1:1 to receive a fixed duration of three cycles lead-in with ibrutinib monotherapy, followed by 12 cycles venetoclax-ibrutinib or chlorambucil-obinutuzumab for six cycles. At a median follow-up of 27.7 months, PFS was significantly longer with venetoclax-ibrutinib than with chlorambucil-obinutuzumab (hazard ratio, 0.216; 95% confidence interval [CI], 0.131 to 0.357; *p* < 0.001). The improvement in PFS with VI was consistent across all predefined subgroups, including patients aged 65 years or older or with a CIRS score greater than 6 or creatinine clearance less than 70 mL/min. With a median follow-up of 34.1 months, PFS remained superior for VI [33••].

**Fig. 1:**
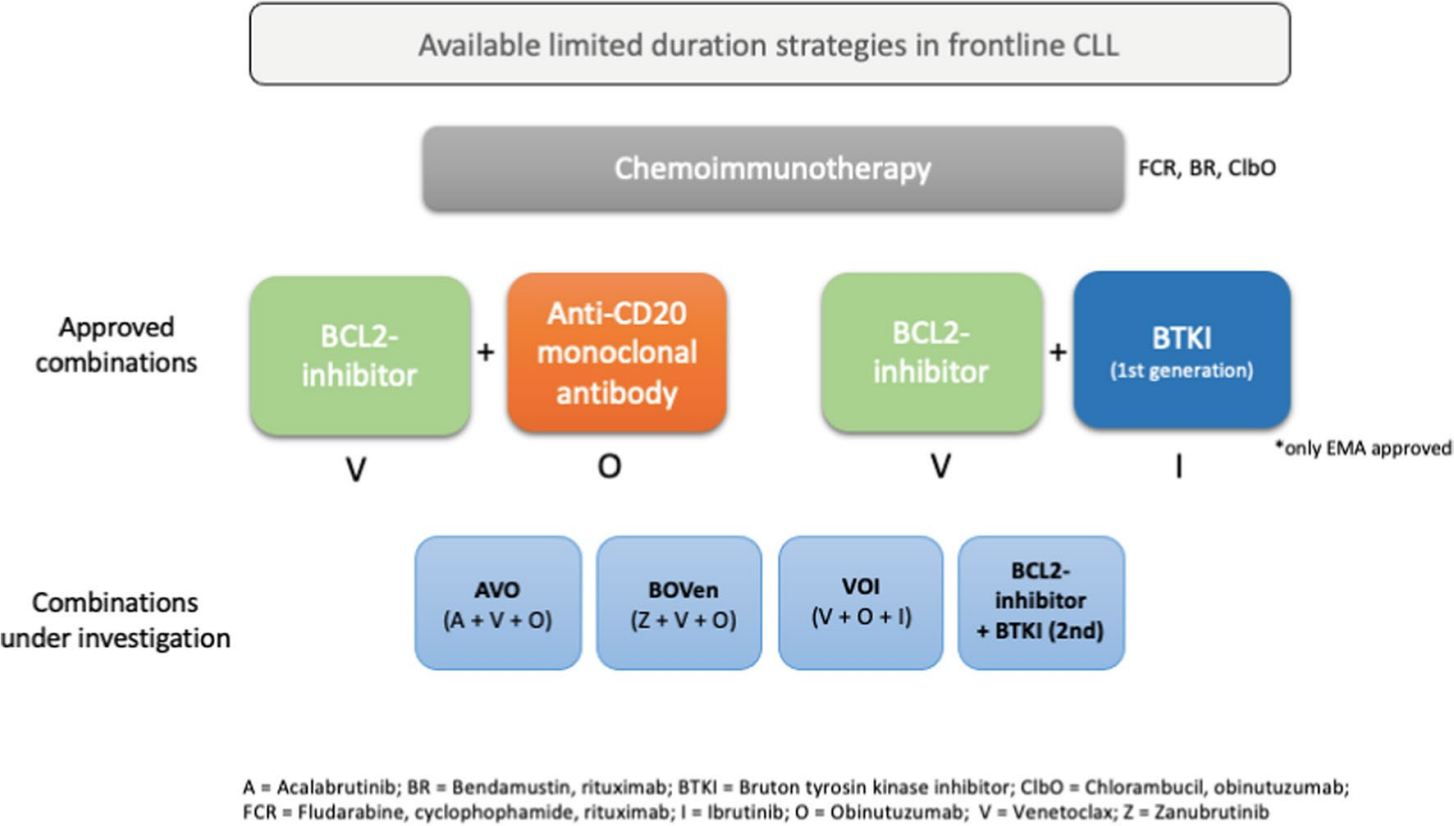
Limited-duration treatment strategies for treatment-naïve CLL

The combination of venetoclax plus ibrutinib and venetoclax plus obinutuzumab respectively is highly effective and offers a fixed-duration treatment for patients with CLL. Figure 1 provides an overview of the available time-limited therapies for first-line treatment of CLL and new treatment options that are still being evaluated.

## The Role of MRD in First-Line CLL Treatment

Clinical response assessment is an essential part of CLL management and can provide information on whether patients have achieved a complete remission (CR) or a partial remission (PR) according to the iwCLL criteria [34]. A conventional response assessment according to iwCLL guidelines requires a physical examination and computer tomography (CT) scan as well as a peripheral and bone marrow blood count. A CR, PR, or stable disease (SD) status is associated with differential outcomes and is a strong post-treatment prognostic factor [2]. It should be noted though that the definitions of CR and PR are somewhat arbitrary in that some cutoffs, such as lymph node size of 1.5 cm, are not necessarily biological defined and therefore might have limited clinical implications; measurement of MRD levels offer an additional way to objectively quantify the depth of remission in the peripheral blood (PB) or bone marrow (BM) after treatment exposure. The most commonly used method in CLL is based on multiparameter flow cytometry, but methods using quantitative real-time PCR or next-generation sequencing (NGS) of the immunoglobulin heavy-chain variable gene (IGHV) can provide equally specific and more sensitive ways to quantify MRD [35–37].

First MRD measurements were already performed under chemotherapy (CT) and showed, despite limited sensitivity (low sensitivity 2 color flow cytometry; MRD levels below 10^–1^), that patients with dMRD after therapy had a worse long-term prognosis than MRD-negative despite achieving complete remission [38]. Clinical trials investigated the effect of the dMRD after treatment on disease development and MRD measurement has rapidly become a prognostic tool, although it took until 2016 for the EMA to declare it a surrogate endpoint in clinical trials.

Fludarabine, bendamustine, and chlorambucil are the most commonly used and investigated chemotherapeutic agents for CLL. The CLL11 study of the German CLL Study Group (GCLLSG) showed that the addition of monoclonal antibodies to CT achieved deeper remission and uMRD in previously untreated patients who received rituximab (ClbR) or obinutuzumab (ClbO) in addition to chlorambucil. The rate of uMRD was 38% for ClbO in contrast to 0% in patients with Clb-mono and 2% with ClbR [39, 40]. These remissions also translated into longer PFS: Obinutuzumab resulted in a significant improvement in PFS (26.7 months) compared to rituximab (14.9 months) when both were combined with chlorambucil (Clb-mono PFS 11.1 months). Longer PFS with chlorambucil plus obinutuzumab is also associated with longer overall survival [41].

Similar results were shown for patients treated with first-line FCR. In the first phase 2 trial testing the FCR first-line regimen, uMRD was observed in 30% of patients at the end of treatment [42]. In the randomized CLL8 study of the GCLLSG, FCR was compared with FC, showing higher uMRD-rates in the FCR arm than in the FC arm (22% vs. 12%). With a median follow-up of 5.9 years, median PFS were 56.8 for FCR and 32.9 months for the FC group [1, 4]. A comparison of FCR with bendamustine/rituximab (BR) in the CLL10 trial showed that FCR achieved higher uMRD rates than BR (49% vs. 38%), which also resulted in longer PFS (55.2 months vs. 41.7 months) [43].

The value of the MRD status as a surrogate endpoint for PFS and OS has been widely demonstrated for CIT: Patients with uMRD after treatment have a better outcome, regardless of whether they achieve partial remission (PR) or complete remission (CR) [10].

The introduction of the Bruton tyrosine kinase inhibitor (BTKI) ibrutinib marks the beginning of targeted therapy; unlike chemotherapies, BTKIs specifically inhibit the B-cell antigen receptor (BCR). While CIT was a fixed-duration treatment (e.g., administered over 6–12 cycles), the BCR inhibitors were developed as continuous therapy until disease progression. In contrast to chemotherapy, targeted therapy with ibrutinib did not result in a high MRD response, and fewer than 10% of patients achieved uMRD with continuous administration [44, 45]. The ECOG1912 study conducted with fit untreated patients without 17p-deletion investigated the comparison between 6 months of FCR and the continuous treatment of ibrutinib + rituximab (IR). Patients receiving ibrutinib for 1 year had a rate of only 5% while patients in the FCR arm achieved a uMRD rate of 57% [45]. Despite not achieving uMRD with ibrutinib, continuous therapy resulted in significantly better PFS and OS compared to FCR. BTKIs as a continuous regimen are thus able to successfully modify CLL and keep the disease under control without achieving a deep molecular remission. For second-generation BTKIs, the rates are similar, with a short follow-up period so far [46, 47].

The BCL2 inhibitor venetoclax was initially used in relapsed/refractory patients as continuous monotherapy and achieved uMRD rates comparable to chemo-/chemoimmunotherapy [14, 48]. The rates were higher when venetoclax was combined with CD20 antibodies like rituximab or obinutuzumab. Patients with uMRD had the longest PFS regardless of whether they achieved a CR or PR [17, 49].

In the first-line setting, the CLL14 trial showed that combining the BCL2 inhibitor venetoclax with the CD20 antibody obinutuzumab induced deep remissions in patients with previously untreated CLL and coexisting comorbidities.

The fixed combination resulted in uMRD rates determined by ASO-PCR in 76% of patients, compared to 35% uMRD with chlorambucil-obinutuzumab [20••]. Patients who had uMRD at the end of treatment had longer PFS compared to patients with dMRD, regardless of treatment arm; the same was observed for overall survival. Conversion from uMRD (< 10^–4^) to re-detectable residual disease (> 10^–4^) was also investigated in the study: The median time to MRD conversion was 21.1 months in the VO group and 6.0 months in the ClbO group (HR 0.36, 95% CI 0.26–0.48) [18]. However, patients with detectable BM-MRD at the end of treatment had a similar short MRD conversion time in both arms, which was also reflected in PFS.

Initial data from the CLL13 trial show similarly high uMRD rates for venetoclax-based treatments in the first-line setting. One of the two co-primary endpoints of the study was uMRD (< 10^–4^), which was determined by flow cytometry in peripheral blood after 15 months. At this time point, the percentage of patients with uMRD was significantly higher in the VO group (86.5%) and the VOI group (92.2%) than in the chemoimmunotherapy group (52.0%) [50]. In patients treated with venetoclax-rituximab, the uMRD rate at 15 months was 57.0%, highlighting the important role of the monoclonal antibody obinutuzumab in MRD eradication. The triple combination of venetoclax, obinutuzumab, and ibrutinib showed better uMRD rates and PFS trend than the two-drug combination in patients with unmutated IGHV status; however, this comparison was not powered and evidence is still needed to confirm a possible advantage of the triple combination in this patient population.

The addition of ibrutinib to venetoclax has also led to high uMRD rates: In the GLOW study at a median follow-up of 34.1 months, patients treated with ibrutinib + venetoclax achieved higher rates of uMRD and deeper (< 10^−5^) responses, with higher concordance between BM and PB, compared to patients treated with chlorambucil + obinutuzumab [33••]. Using stricter cutoffs (< 10^–4^ or < 10^–5^), combination VI showed high concordance in uMRD levels between PB and BM (90.9–92.9%) and 97.8% of patients who had a uMRD < 10^–5^ in PB had a uMRD < 10^–4^ in BM [51•]. MRD rates 3 months after end of treatment (EOT) were 54.7% for VI and 39.0% for ClbO. For VI, uMRD rates tended to be higher in patients with unmutated IGHV status compared to mutated status (59.7% vs. 40.6%). VI achieved higher uMRD rates than ClbO for all relevant mutations [52••]. With u-MRD rates of 46.2% after six cycles of VI and finally 54.7% 3 months after end of therapy, MRD eradication was most effective at the start of the therapy. Post-treatment, 77.6% of patients in the VI arm maintained their uMRD status to EOT + 18, compared to 12.2% in the ClbO arm. MRD kinetics and sustained response show strong efficacy of the fixed-duration VI combination in elderly patients with high-risk genomic features.

In the CAPTIVATE trial, patients of the MRD cohort and the fixed-duration cohort respectively received three cycles of ibrutinib lead-in then 12 cycles of combination VI. Patients of the MRD cohort were then randomized based on MRD status to receive either further treatment or placebo. Before randomization, the highest uMRD rates were 75% in PB and 68% in BM [53]. Patients with uMRD were randomly assigned to either ibrutinib or placebo, with 2-year disease-free survival rates after randomization of 95% and 100%, respectively, without statistical significance. Patients who did not achieve uMRD after 12 cycles were randomized either to ibrutinib or the combination (VI). As a result, significant improvements in uMRD and CR/CRi rates were observed with the combination than with ibrutinib alone. Another year of treatment, however, did not lead to further improvement [54••]. The CAPTIVATE trial demonstrated that an MRD-guided extension of the treatment provides an improvement for patients with dMRD and deep remission can be achieved with the combination of ibrutinib and venetoclax. Table 1 provides an overview of the results of the previously discussed pivotal studies.

**Table 1:** Trial outcomes of time-limited therapies in the first-line setting

Trial	Regimen	PFS	OS	uMRD PB (< 10^–4^)	uMRD BM (< 10^–4^)
CLL14	ClbO (*n* = 216)	Median PFS 36.4 m 49.5% at 39.6 m 27.0% at 65.4 m	87% at 39.6 m 77% at 65.4 m	EOT + 3 m 35% EOT + 18 m 7%	EOT + 3 m 17.1%
	VO (*n* = 216)	Median PFS NR 81% at 39.6 m 62.6% at 65.4 m	87% at 39.6 m 81.9% at 64.4 m	EOT + 3 m 76% EOT + 18 m 47%	EOT + 3 m 56.9%
CAPTIVATE FD	VI (*n* = 159)	95% at 24 m 88% at 36 m 79% at 50 m	98% at 24 m 98% at 36 m 98% at 50 m	Best uMRD 77%	Best uMRD 60%
GLOW	ClbO (*n* = 105)	44.1% at 24 m 35.8% at 30 m	88.6% at 27.7 m	EOT + 3 m 39% EOT + 18 m 5%	EOT + 3 m 17.1%
	VI (*n* = 106)	84.4% at 24 m, 80.5% at 30 m	90% at 27.7 m	EOT + 3 m 54.7% EOT + 18 m 42%	EOT + 3 m 51.9%
CLL13	CIT- FCR/BR (*n* = 229)	Median PFS 52 m 75.5% 38.8 m	95% at 38.8 m	52% at 15 m	37.1% at 15 m
	VR (*n* = 237)	Median PFS 52.3 m 80.8% at 38.8 m	96.5% at 38.8 m	57% at 15 m	43% at 15 m
	VO (*n* = 229)	Median PFS NR 87.7% at 38.8 m	96.3% at 38.8 m	86.5% at 15 m	72.5% at 15 m
	VOI (*n* = 231)	Median PFS NR 90.5% at 38.8 m	95.3% at 38.8 m	92.2% at 15 m	77.9% at 15 m

*OS* overall survival; *PFS* progression-free survival; *uMRD* undetectable minimal residual disease; *PB* peripheral blood; *BM* bone marrow; *NR* not reached; *FD* fixed duration; *m* month; *BR* bendamustine, rituximab; *CIT* chemoimmunotherapy; *ClbO* chlorambucil, obinutuzumab; *FCR* fludarabine, cyclophosphamide, rituximab; *I* ibrutinib; *O* obinutuzumab; *V* venetoclax

The long-term outcomes of targeted CLL therapies with fixed treatment duration in terms of durability of remissions and survival are still unknown. While the majority of patients achieve uMRD in the peripheral blood at the end of treatment, a subset of patients with detectable MRD levels show limited response to treatment. The biological causes of MRD response (i.e., detectable MRD levels ≥ 10^–4^) are not yet clear.

## Using MRD to Guide Time-Limited Treatment

Clinical trials currently investigate different approaches to guide treatment with targeted agents based on MRD. The main differences between the approaches include different time-point of the MRD assessment, the evolving measurement methods, and the criteria to terminate or continue treatment.

One approach that the GCLLSG has already evaluated in several phase 2 trials is MRD-guided maintenance therapy: Patients who had uMRD in PB and CR at two follow-up visits were able to discontinue maintenance therapy [55–57]. In the CLL2-BAG study, 87% of treatment-naive/refractory-relapsed patients treated with venetoclax/obinutuzumab after bendamustine debulking achieved non-measurable MRD rates, and the majority of patients were able to discontinue maintenance therapy at the earliest possible time point.

The CLARITY trial introduced a new approach to disease control that takes into account the individual time to reach uMRD [28]. Patients who achieved uMRD within 6 months received additional 6 months of therapy, in total 12 months of venetoclax and ibrutinib, while those with 12 months to achieve uMRD in the BM received a total of 24 months of treatment. After 6 months of treatment, 24% of patients achieved uMRD and received 12 months of treatment; after 12 months, 58% of patients were MRD negative and were treated for a total of 24 months. This concept initially explored in a phase 2 study in relapsed/refractory patients will be further investigated in the phase 3 FLAIR study in a treatment-naive population [58].

The single-arm phase 2 BOVen trial investigated an MRD-guided treatment strategy in therapy-naive CLL/SLL patients [59]. The treatment consists of oral zanubrutinib, intravenous obinutuzumab, and venetoclax and was discontinued after 8–24 cycles if the predefined criteria for uMRD (≤ 10^–4^ evaluated by flow cytometry) in PB/BM were met. With a median follow-up of 40 months (4.1–47.4) and treatment duration of 10 cycles (IQR 8–14), 96% of the patients had uMRD in PB; 92% had uMRD in PB and BM after a median of 8 months (IQR 6–11.5). Long-term follow-up of BOVen demonstrates high rates of durable uMRD. BOVen was well tolerated and 89% met its primary endpoint uMRD in PB and BM. These data support further evaluation of the BOVen regimen.

A further MRD-guided phase 2 trial is the AVO triplet regimen [60]. The unselected baseline population of previously untreated patients was supplemented by an expansion of patients with *TP53* aberrant disease. Triple combination of acalabrutinib, venetoclax, and obinutuzumab showed a durable response; however, patients with a high-risk *TP53* mutation did not respond as well. The primary end point (uMRD measured by flow cytometry) assessment was taken on C16D1: For patients who achieved a BM uMRD and a complete response (CR) per iwCLL criteria, therapy was discontinued. Patients with a partial response (PR) would continue treatment with nine cycles of acalabrutinib and venetoclax and a second assessment took place on C25D1. If uMRD was not achieved at this point, acalabrutinib and venetoclax were continued until progression or toxicity. The median follow-up time was 27.6 months and at c16d1, 38% of participants had a complete remission with uMRD in the bone marrow. In general, patients with high-risk genetics had lower uMRD rates than the overall study population. The primary endpoint of this study was not met. The high proportion of patients with uMRD in the bone marrow suggests further investigation of this strategy, which is being tested in the ongoing phase 3 AMPLIFY trial [61]. Another question that this trial aims to address is whether a time-limited treatment with acalabrutinib in combination with other drugs is safe and effective.

Currently, there is no consensus on the use of MRD as a prognostic tool and several challenges must be addressed before it can be widely used in routine practice. First of all, the measurement method needs to be standardized. While the threshold of 10^–4^ provides good comparability with long-term clinical outcomes of chemo-based therapies, lower response values are informative for depth of remissions and allow serial tracking of response kinetics (MRD conversion). Upcoming investigations of new targeted treatment combinations in clinical trials should further explore the potential impact of deeper response rates. To be relevant to standard of care and especially MRD-guided therapy, MRD methods that meet regulatory standards and are equally available must be used for comparability. As a second point, the ideal MRD compartment needs to be defined. CLL is a multicompartment disease that can affect not only the bone marrow and blood, but also other lymphoid and extra-lymphoid tissues [62, 63]. Certain agents, particularly anti-CD20 antibodies, preferentially eliminate cells in the peripheral blood and are less effective in other compartments [64]. Several studies have confirmed that CR or PR of the bone marrow is not very relevant in the presence of uMRD, and the outcome is not entirely clear in patients with inconsistent MRD results in PB and BM. To address these issues, attempts are being made to use cell-free DNA from plasma to measure MRD to provide information about residual disease in different compartments [65, 66]. This assay also allows tracking and responding to the emergence of new clones and potential mutations. This leads indirectly to the third point: the quality of MRD remission must be taken into account, as not all patients with uMRD remain in remission for the same length of time. In particular, patients with a high-risk constellation have a shorter time to the next treatment. This suggests that genetic factors play a role in the stability of remission [65, 67].

## Ongoing Studies

The combination of targeted agents has paved the way for the development of therapeutic regimens that achieve deep molecular remissions and thus the possibility to discontinue therapy. A key issue to be addressed is whether targeted time-limited therapies with high uMRD rates or continuous therapy with BTK inhibitors, which rarely results in negative MRD remissions, are beneficial for CLL patients. The CLL17 study of the GCLLSG (NCT04608318) addresses this very important question: Patients with previously untreated CLL are randomized to either continuous ibrutinib monotherapy or fixed-duration venetoclax-obinutuzumab or venetoclax-ibrutinib (1:1:1). The question of whether molecular and cytogenetic risk characteristics have an impact on treatment might be answered with subgroup analyses in this heterogeneous patient collective (unmutated/mutated IGHV, del(17p)/*TP53*). MRD assessments are performed in CLL17 at fixed time points.

The FLAIR trial (ISRCTN01844152) was designed as a two-arm study comparing FCR and IR in a previously untreated patient population without *TP53* aberrations [58]. In 2017, the ongoing FLAIR trial was adapted to add two arms: I monotherapy and VI [68]. The duration of therapy in both arms was determined by MRD status, with MRD assessment in PB and BM at various time points. If PB is MRD negative, it is retested after 3 months, and if still negative, PB and BM MRD are assessed 3 months later. If both are MRD negative, the initial MRD-negative PB result is considered the time to MRD negativity, and therapy continues for twice that period, allowing patients to potentially stop therapy earliest at 2-years post-randomization. The maximum duration of therapy is 6 years.

Another ongoing trial of the GCLLSG, CLL 16 (NCT05197192), will only enroll previously untreated patients with high-risk CLL: The phase 3 trial will compare VO to VO plus acalabrutinib in patients with a 17p deletion and/or *TP53* mutation and/or complex karyotype. In the triplet arm, patients achieving dMRD after cycle 14 will continue treatment with acalabrutinib for up to 24 cycles.

The randomized phase III MAJIC trial (NCT05057494) is another first-line study investigating the optimal duration of finite treatment [69]. In both treatment arms (acalabrutinib-venetoclax vs. venetoclax-obinutuzumab), duration of therapy will be guided by clinical response in addition to MRD status. The aim is to enroll 600 patients in the study, including those with a high-risk genetic profile.

Further ongoing trials are currently testing double/triple regimes consisting of BTKI and BCL-2 inhibitors with or without monoclonal antibodies. Table 2 gives an overview of further ongoing clinical trials with fixed duration in first-line setting.

**Table 2:** Ongoing clinical trials

Study	Trial population	Study treatment	Primary endpoint
CLL17 (NCT04608318)	*N* = 897 ≥ 18 y Fit/unfit No aberrations excl	I: until progression VO: 12 months VI: 15 months; venetoclax 12 months	PFS
FLAIR (ISRCTN01844152)	*N* = 1516 ≤ 75 y Fit/ eGFR > 30 mL/min del (17p) < 20%	IR → I*: until progression VI: flexible duration according to MRD CIT: FCR 6 cycles *IR replaced by I mono in 2018	PFS
AMPLIFY (NCT03836261)	*N* = 780 ≥ 18 y Fit/ *TP53* aberrations excl	AV: 15 months, venetoclax 12 months AVO: 15 months, venetoclax 12 months CIT: FCR/BR 6 cycles	PFS
MAJIC (NCT05057494)	*N* = 600 ≥ 18 y Fit/ unfit No aberrations excl	AV: 15 months; Ven 12 months VO: 12 months (dMRD after 12 months venetoclax = additional 12 months treatment)	PFS MRD-guided AV/VO
CRISTALLO (NCT04285567)	*N* = 165 ≥ 18 y Fit/ *TP53* aberrations excl	VO: 12 months CIT: FCR/BR 6 cycles	MRD BM at month 15
ECOG-ACRIN EA9161 (NCT03701282)	*N* = 720 18– 69 y del (17p) excl	IO: until progression VOI: 19 months; venetoclax 12 months	PFS
FILO ERADIC (NCT04010668)	*N* = 120 ≥ 18 y Fit/ *TP53* aberrations excl	VI: 15 or 27 months according to MRD CIT: FCR 6 cycles	MRD BM at month 27
CLL 16 (NCT05197192)	*N* = 178 ≥ 18 y del(17p) and/or *TP53* mutation and/or complex karyotype	AVO:15 months, venetoclax 12 months VO: 12 months (dMRD after 14 cycles AVO = additional 12 cycles treatment with acalabrutinib)	PFS

*OS* overall survival; *PFS* progression-free survival; *MRD* minimal residual disease; *dMRD* detectable minimal residual disease; *BM* bone marrow; *A* acalabrutinib; *BR* bendamustine, rituximab; *CIT* chemoimmunotherapy; *FCR* fludarabine, cyclophosphamide, rituximab; *I* ibrutinib; *O* obinutuzumab; *V* venetoclax

## Practical Implications of MRD

Given the large body of evidence demonstrating the correlation between end-of-treatment MRD and long-term clinical outcomes, MRD can be considered an informative biomarker in routine clinical care. Multiple studies have demonstrated that patients who remain MRD-positive > 10^–4^ in peripheral blood have a high risk of early relapses within 2–3 years after treatment [20••, 33••, 70]. Hence, testing of MRD in peripheral blood at the end of treatment by flow cytometry can provide valuable information in some patients for the sake of prognostication. Since so far there is no randomized evidence suggesting a benefit of MRD-guided treatment extension, the MRD status should not be used to modify treatment outside of clinical studies. Likewise, serial MRD assessments currently do not have clinical implications in routine clinical care, as the decision to treat a progressive CLL should be guided by iwCLL criteria for treatment indication [34].

## Conclusion

Time-limited therapy has become one of the cornerstones of modern CLL management, with virtually all novel agents being explored in a potentially time-limited fashion.

For routine clinical care, time-limited therapy is currently synonymous to fixed-duration therapy, since a pre-defined number of treatment cycles are administered to patients, regardless of the remission depth and quality. By measuring MRD, post-treatment response can be objectively assessed and thereby valuable prognostic information on duration of response and survival can be retrieved. Based on these insights, individualized treatment duration and intensity based on MRD status has been widely explored in phase 2 studies. However, in order to further establish MRD in clinical routine and beyond clinical studies, randomized studies are warranted to demonstrate whether MRD-guided, time-limited treatment provides improved outcomes over fixed-duration or continuous treatment of patients with CLL.
